# Genomic epidemiology unravels simultaneous clusters of linezolid-resistant *Staphylococcus epidermidis* isolated in Austria, 2023–2025

**DOI:** 10.3389/fmicb.2026.1934085

**Published:** 2026-10-20

**Authors:** Adriana Cabal Rosel, Heidrun Kerschner, Johannes Weinberger, Anna Stöger, Kathrin Lippert, Beatriz Daza Prieto, Alexander Mellmann, Petra Apfalter, Werner Ruppitsch, Rainer Hartl

**Affiliations:** 1 Institute for Surveillance and Infectious Disease Epidemiology, Austrian Agency for Health and Food Safety, Vienna, Austria; 2 Institute for Medical Microbiology and Hygiene, Austrian Agency for Health and Food Safety, Vienna, Austria; 3 National Reference Center for Antimicrobial Resistance, Linz, Austria; 4 Ares Genetics GmbH, Vienna, Austria; 5 Institute of Hygiene, University Hospital Muenster and University of Muenster, Muenster, Germany; 6 Institute of Hygiene and Medical Microbiology, Medical University Innsbruck, Innsbruck, Austria; 7 Medical Faculty, Johannes Kepler University Linz, Linz, Austria

**Keywords:** antimicrobial resistance, genomic clusters, linezolid, *Staphylococcus epidermidis*, whole-genome sequencing

## Abstract

**Introduction:**

*Staphylococcus epidermidis* is an opportunistic pathogen causing bloodstream and device-associated infections. Due to the use of linezolid (LZD) against infections caused by multidrug-resistant Gram-positive bacteria, the emergence of LZD-resistant *S. epidermidis* (LRSE) is an increasing concern. This study aimed to characterize genomic clusters, resistance mechanisms, and epidemiological links among LRSE isolates collected during a suspected outbreak in two Austrian hospitals.

**Methods:**

A confirmed case was defined as a patient with LRSE (MIC > 4 mg/L) isolated from clinical or screening samples in Hospital A or Hospital B between March 2023 and April 2025. All isolates underwent whole-genome sequencing (WGS) for typing and identification of resistance determinants. Epidemiological data, including the hospital and ward of detection as well as inter-hospital transfers, were analyzed to explore potential epidemiological links between cases.

**Results:**

A total of 53 LRSE cases were identified, including 22 in Hospital A and 31 in Hospital B. A LZD-resistant *Staphylococcus hominis* isolate originating from a patient admitted to Hospital B after traveling abroad and no epidemiological link with the LRSE cases was also recovered. Epidemiological and genomic data identified five clusters belonging to three sequence types (ST2, ST5, ST22). Most Cluster 1 cases were detected in Hospital B, especially in the surgical ICU, while Clusters 2–4 were exclusively identified in Hospital A. However, the available epidemiological information was insufficient to determine transmission direction or identify the origin of the clusters. Forty-five of 53 LRSE isolates displayed high-level LZD resistance (MIC ≥48 mg/L). Distinct resistance-associated mutations were identified in each ST, including G2576U in ST2 isolates, C2534U and U2504A in ST22 isolates, and characteristic alterations in ribosomal proteins L3 and L4.

**Conclusion:**

Genomic analysis identified five distinct LRSE clusters belonging to three STs and revealed ST-specific resistance mutations, suggesting multiple independent evolutionary pathways to LZD resistance. However, limited epidemiological information, including incomplete data on LZD administration and patient movements, prevented definitive assessment of transmission dynamics. Our findings demonstrate the utility of WGS for the detection and characterization of LRSE clusters and highlight the importance of comprehensive epidemiological data for interpreting genomic links.

## 1 Introduction

*Staphylococcus epidermidis* (*S. epidermidis*) is a Gram positive, coagulase-negative bacterium often found on the skin of healthy humans (Lee and Anjum, 2025). Despite its role as a commensal organism, *S. epidermidis* can act as an opportunistic pathogen, particularly in immunocompromised patients, such as those in intensive care units (ICUs), and is therefore frequently implicated in nosocomial infections. *S. epidermidis* is able to form biofilms, hence it can colonize intravenous catheters, surgical wounds and it is one of the leading causes of bloodstream infections (BSIs) in hospital settings (Siciliano et al., 2023). In addition, the treatment of such infections is often complicated by a high prevalence of antimicrobial resistance, particularly to beta-lactam antibiotics such as methicillin.

Linezolid (LZD), an oxazolidinone-class antibiotic, is a critical last-resort therapeutic option for infections caused by *S. epidermidis* and other beta-lactam resistant gram-positive bacteria, including vancomycin-resistant Enterococci (VRE) (Mazuski et al., 2017). However, in the last decade, LZD resistance (minimum inhibitory concentration [MIC] ≥ 8 mg/L) has become common, leading to the emergence of LZD-resistant *S. epidermidis* strains (LRSE), many of which caused hospital outbreaks (Huber et al., 2021). One of the primary drivers of the increase in LZD-resistant *S. epidermidis* (LRSE) via selective pressure is prior LZD exposure in patients (Seedat et al., 2006). In addition, evidence showed that LRSE can be transmitted between patients through healthcare workers, highlighting the critical importance of proper hand hygiene in hospital settings (Lépine et al., 2024).

*S. epidermidis* belonging to multilocus sequence type (ST) ST2, ST5 and ST22 seem to be prevalent worldwide and are often associated to hospital outbreaks (Cinthi et al., 2024; Dortet et al., 2018). The main mechanism of LZD resistance in *S. epidermidis* in the hospital environment is the emergence of point mutations in the domain V of the 23S rRNA gene. One or more 23S rRNA gene copies can be mutated and there is an association between the MIC and the number of mutated copies. Mutations such as U2504A and C2534U have been associated with high-level LZD resistance when present in multiple gene copies (Pournaras et al., 2013). Point mutations in the 50S ribosomal proteins, particularly L3, L4, and L22, also contribute to LZD resistance, although at lower level. During prolonged antibiotic therapy with LZD, spontaneous genomic changes in the 23S rRNA and 50S proteins can frequently occur. In addition to point mutations, other mechanisms of LZD resistance include plasmid-encoded genes, such as *cfr*, *cfr*(D), *optr*A, and *poxt*A (Weßels et al., 2018; Côrtes et al., 2022). These are all inter-species transferable genes often associated with high-level resistance, in particular *cfr*, and although less common than the point mutations, they have been found not only in hospital settings but also in animals and the environment (Locke et al., 2010). Horizontal gene transfer has been described in the animal gut, especially in pigs (Shen et al., 2024). However, prolonged antibiotic exposure continues to be the main driver of LZD resistance. No matter the mechanism of resistance, addressing LZD resistance is essential for managing infections caused by *S. epidermidis* and accurate diagnostic methods are needed to prevent the dissemination of resistance. In addition, the dual nature of *S. epidermidis*, serving both as commensal and as pathogen, underscores the need for genomic surveillance especially in hospital environments.

A previous study conducted in an Austrian hospital reported the increase of LZD resistance and the presence of different LRSE clones between 2011 and 2019 (Huber et al., 2021). However, little is known about the presence of LRSE in other hospitals in the country.

Between March and April 2023, three LRSE isolates were detected in blood cultures from patients staying at two neighboring hospitals in Austria, where frequent patient transfers and shared clinical care were common. As LRSE detection in blood samples was rare in those hospitals, this triggered outbreak suspicion. As cases continued to appear in both hospitals, WGS was initiated to detect possible genomic clusters, elucidate the underlying resistance mechanisms in the isolates, as well as to assess potential epidemiological links based on patient locations and inter-hospital transfers.

## 2 Methods

### 2.1 Cases and isolates

Laboratory-based surveillance of LRSE isolates was performed from March 2023 to April 2025, after which no further isolates were submitted to our laboratory. A case was defined as a patient from whom a LRSE isolate was recovered between March 2023 and April 2025 at hospital A or hospital B, located in the same region of Austria. Only the first recovered LRSE isolate per patient was included. Samples originated either from clinical specimens for routine bacterial culture or from dedicated surveillance samples investigated for the presence of LRSE. Screening samples included nasal swabs, throat swabs, rectal swabs, skin swabs (“Z-swabs”. Z-swabs are combined axillary swabs, umbilical and inguinal swab) and urine samples collected on admission. Linezolid resistance was defined either by a resistant result obtained by disk diffusion testing or by a linezolid MIC >4 mg/L, according to EUCAST breakpoint tables (v 13.1, 2023 and v 14.0, 2024). The study period reflected the complete timeframe during which isolates were submitted to our laboratory. Anonymized demographic data, clinical characteristics, microbiological details, antimicrobial therapy and information on the resistance phenotype of *S. epidermidis* to selected antibiotics were available for most LRSE cases.

During the investigation period, one linezolid-resistant *Staphylococcus hominis* (LRSH) isolate from an imported case was also identified in Hospital B. As it did not meet the LRSE case definition, the isolate was analyzed separately and it was not included in the cluster analysis.

All patient data used in this scientific work were anonymized. The study was approved by the Ethics Committee of the Faculty of Medicine, Johannes Kepler University Linz (1152/2023).

LZD resistance was detected initially via disk diffusion according to EUCAST. In the further course of the suspected outbreak, the chromogenic screening medium CHROMAgar LIN-R (CHROMagar, France) was used according to the manufacturer’s instructions for screening of different body locations (nasal swab, Z-swab on admission to the most severely affected wards. Antimicrobial susceptibility testing (AST) was performed on all isolates using disk diffusion according to EUCAST breakpoint tables (v 13.1, 2023 and v 14.0, 2024) also for oxacillin (determined by cefoxitin-screening), clindamycin, erythromycin, tigecycline, doxycycline, fusidic acid, rifampicin, and trimethoprim-sulfamethoxazole screening (EUCAST, 2024). Additionally, gentamicin and vancomycin susceptibility were tested via disk diffusion in 15 and eight LRSE isolates, respectively, according to different clinical needs. MICs for LZD were obtained for all LRSE isolates using gradient diffusion testing (Etest, bioMerieux) as described by the manufacturer. Additionally, MICs for vancomycin and teicoplanin were determined for eight and five isolates, respectively, by broth microdilution (UMIC, Bruker, Germany).

### 2.2 WGS

For genomic analysis, DNA was extracted from isolated colonies with MagAttract HMW DNA kit (Qiagen, Heidelberg, Germany). Pair-end sequencing (2 × 150 bp) was performed on an Illumina Nextseq2000 using the Nextera XT library preparation kit (Illumina, San Diego, CA, USA) and raw reads assembled as previously described (Cabal et al., 2017). A subset of 22 isolates collected during 2022–2023 was selected for Nanopore sequencing to assess LZD resistance mechanisms potentially overlooked by short-read sequencing, including the genomic context of resistance determinants. The Native Barcoding Kit or Rapid Barcoding Kit (SQK-NBD114.24 and SQK-RBK114.24, Oxford Nanopore Technologies, UK) was used for library preparation, and libraries were sequenced on R10.4.1 flow cells using a GridION device for 72 h. Super-accurate (SUP) basecalling was performed with Guppy v6.1.5, and reads were assembled using Flye v2.9.1. Hybrid assemblies were generated with Unicycler v0.5.0 from Illumina and Nanopore reads.

Sequencing quality was assessed using MBioSEQ Ridom Typer Version 11.1.0 (Bruker, Germany). Quality-control metrics included sequencing depth, assembly size, contig number, N50, proportion of successfully typed cgMLST targets, and contamination screening. A minimum sequencing depth of >35x, >90% of good cgMLST (preferably >95%) targets as well as no contamination, was required for all isolates. Isolates not passing these quality criteria were excluded from downstream analyses analysis.

We used a previously published cgMLST scheme with 1,828 core genes in MBioSEQ Ridom Typer to investigate genetic relatedness between the isolates (Papan et al., 2021). Sequence types (STs) according to the *S. epidermidis* MLST scheme were extracted using the same platform. A minimum spanning tree (MST) was generated using a cluster threshold of ≤6 allelic differences (AD) as previously proposed for LRSE transmission (Campmann et al., 2023). Publicly available LRSE genomes identified through a literature search were analyzed alongside the isolates from this study using the same cgMLST approach, provided that raw sequencing data were available and the isolates belonged to sequence types detected in our study population (Huber et al., 2021; Cinthi et al., 2024; Dortet et al., 2018). Plasmids and other mobile genetic elements as well as the *ic*ACBD operon were extracted using MBioSEQ Ridom Typer.

A Neighbor-Joining tree generated with MBioSEQ Ridom Typer (based on the obtained cgMLST allelic distances) was subsequently imported into Microreact together with a metadata table containing information on the found plasmid replicons, antimicrobial resistance genes (ARGs), virulence-associated genes, sequence type, year of isolation, and cluster assignment. Microreact was used to visualize phylogenetic relationships and associated genomic features.

ARGs including LZD transferable ARGs such as *cfr*, *cfr(D)*, *optrA* and *poxtA* were analyzed with AMRFinderPlus and point mutations associated with LZD resistance in the 50S proteins L3, L4 and L4 and 23S rRNA were investigated with ARIBA using Illumina sequence data (Hunt et al., 2017). Variant frequencies were extracted from the ARIBA 23S rRNA reports using the allele depths reported in the *smtls_nts_depth* field. We only considered variants classified by ARIBA as SNPs and not those variants classified as *HET* (heterozygous). Only 23S rRNA mutations previously associated with LZD resistance were considered in downstream analyses.

For representative isolates with available hybrid genome assemblies, 23S rRNA operons were identified using Barrnap v1.10.5. Individual 23S rRNA gene copies were extracted from the assemblies and screened for resistance-associated mutations to determine their distribution among gene copies.

The sequencing data (both Illumina and Nanopore) produced in this study have been submitted to the Sequence Read Archive (SRA) and are publicly available under the BioProject accession number PRJNA1492009.

### 2.3 Statistical analysis

Statistical analyses to assess differences in LZD MIC across STs were performed in R version 4.5.0. Non-LRSE isolates and isolates with insufficient sequencing quality were excluded from the analysis. Differences between STs were evaluated using the Kruskal–Wallis test, and when significant, pairwise comparisons were performed using Dunn’s test with Bonferroni correction.

## 3 Results

### 3.1 Case description

A total of 53 patients with LRSE were included in this study and met the case definition. Additionally, the LRSH isolate from an imported case was analyzed separately.

The median age of the cases was 60 years (IQR: 14), and 37 (70%) were male (Table 1). Twenty-two LRSE cases were detected in Hospital A and 31 LRSE plus the LRSH in Hospital B. Three cases were initially admitted to Hospital B and later transferred to Hospital A. Aditionally, two cases were first admitted to hospitals other than A or B and subsequently transferred to Hospital A in all five cases, LRSE detection occurred only after the transfer. For all remaining cases, either transfer information was unavailable or no transfer took place. Most patients from Hospital A had been in an oncology ward (50%), whereas patients from Hospital B were primarily in the surgical ICU at the time of detection (55%). Immunosuppression was documented in 20 (91%) Hospital A cases, and hematological-oncological diseases were the most frequent (91%) underlying conditions in those patients. At the time of LRSE detection, 20 Hospital A patients (91%) were receiving antibiotic therapy. Recent exposure to LZD (within 28 days) was confirmed in 7 patients (32%), with a median treatment duration of 9 days (range: 8–14). Patient data are available in Supplementary Table S1.

**Table 1:** Demographic and clinical characteristics of LRSE-positive patients (*n* = 53).

Variable	Value
Age (years), median [IQR]	60 [14]
Sex, *n* (%)	
Male	37 (70%)
Female	16 (30%)
LRSE	
Hospital A	22 (42%)
Hospital B	31 (59%)
Ward type	
ICU	18 (34%)
Oncology	12 (23%)
Surgery	9 (17%)
Other	9 (17%)
Outpatients oncology	5 (9%)
Specimen origin	
Screening isolates*	23 (43%)
Central venous catheter tip	12 (23%)
Wound	10 (19%)
Blood culture	6 (11%)
Other clinical samples	2 (4%)
Transfer from other hospital/LTCF	
Yes	5 (10%)
No	17 (33%)
Unknown	31 (59%)
Hospital contact in previous year, *n* (%)	
Yes	22 (42%)
Unknown	31 (59%)
LZD therapy (previous 28 days), *n* (%)	
Yes	7 (13%)
No	12 (23%)
Unknown	34 (64%)
Duration of LZD therapy, days, median (range) (*n* = 5)	9 (8–14)
Current antibiotic therapy, *n* (%)	
Yes	20 (38%)
Unknown	33 (62%)
Immunocompromised, *n* (%)	
Yes	20 (38%)
No	2 (4%)
Unknown	31 (59%)
Current underlying disease	
Hematology-oncology	20 (38%)
Respiratory disease	2 (4%)
Unknown	31 (58%)

*z swab, rectal, nose or throat swab, stool and urine.

### 3.2 Isolates and susceptibility testing results

From each patient, one LRSE isolate was recovered (*n* = 53). The largest individual specimen category was central venous catheter tips (*n* = 12, 22.6%), while screening specimens collectively accounted for 43.4% of isolates.

Disk diffusion testing demonstrated that all 53 isolates were resistant to oxacillin and LZD and 45 of them exhibited high-level LZD resistance, defined as a MICs ≥48 mg/L (Supplementary Figure S1). High frequencies of resistance were observed for clindamycin (*n* = 46, 87%) and fusidic acid (*n* = 35, 66%). Trimethoprim-sulfamethoxazole resistance was observed in 29 (55%) isolates, while resistance to erythromycin and rifampicin was detected in 23 (43%) isolates. Low frequencies of resistance were observed for doxycycline (*n* = 2, 4%) and tigecycline (*n* = 1, 2%). Gentamicin resistance was detected in all 15 isolates tested. Regarding glycopeptides, all eight isolates tested for vancomycin were found susceptible with broth microdilution, and four out of five isolates tested were susceptible to teicoplanin also with broth microdilution. The LRSH isolate presented a MIC of 64 mg/L.

### 3.3 Genomic epidemiology

All study isolates but one were successfully sequenced. One LRSE isolate failed sequencing quality control and was excluded. WGS-based typing identified three STs among the 52 LRSE isolates that passed quality filters: ST2 (*n* = 22; 42%), ST5 (*n* = 3; 6%), and ST22 (*n* = 27; 51%). The LRSH isolate was assigned to ST2 using the species-specific MLST scheme available in MBioSEQ (Figure 1).

**Figure 1:**
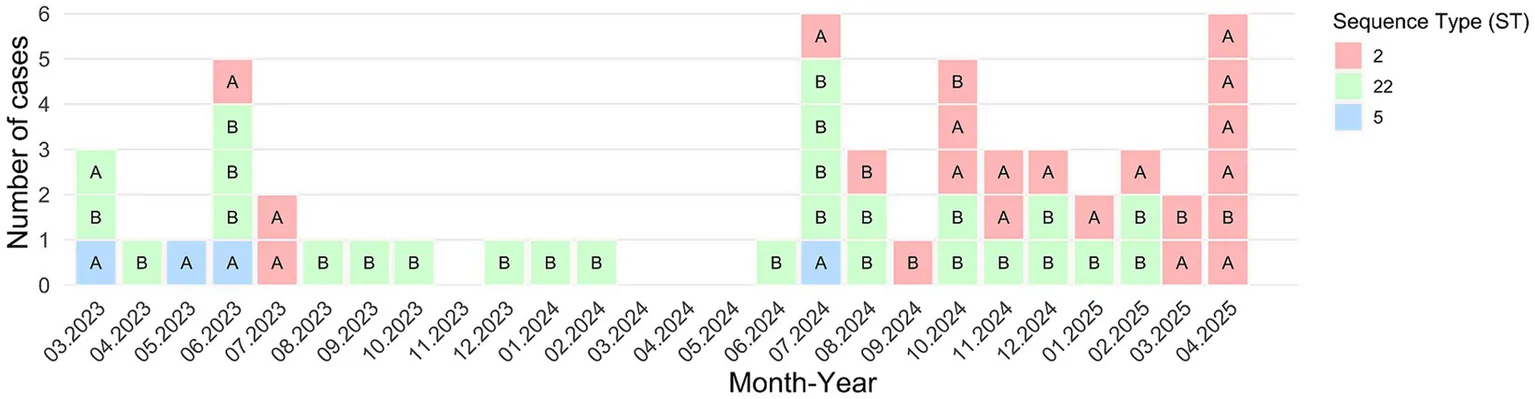
Epicurve showing the 53 LRSE cases included in this study, Austria, 2023–2025. Each box represents a single case and it is labeled by hospital (A or B). Colors represent Sequence types.

Kruskal–Wallis tests revealed statistically significant differences between STs regarding LZD MIC values (*p* = 2.2 × 10^−6^). Post-hoc pairwise comparisons (Dunn test with Bonferroni correction) indicated that ST22 exhibited significantly higher MICs than both ST2 (p_adj = 1.1 × 10^−5^) and ST5 (p_adj = 0.0064), whereas no significant difference was found between ST2 and ST5 (p_adj = 1.00) (Supplementary Figure S1).

WGS-based typing revealed five clusters, three in Hospital A and two in Hospital B, as well as eight singletons:

Cluster 1 (ST22) spanned a period of two years, with pairwise isolate distances ranging from 0 to 6 AD. It was the largest cluster, comprising 18 clinical and 7 screening isolates, respectively (Figure 2). Of these, 24 cases were detected in Hospital B, while one case (Pt02) was identified in Hospital A. For Pt02, it was confirmed that although the patient had not been hospitalized in Hospital B, the patient underwent three diagnostic procedures in Hospital B between December 2022 and January 2023, preceding the LRSE isolation in March 2023 at Hospital A. This case was also the only individual within Cluster 1 for whom linezolid therapy was documented, with a treatment duration of eight days. Two other cases within the cluster 1 (Pt31 and Pt40), as well as one additional case whose isolate was only 9 allelic differences (AD) from Cluster 1 (Pt17), had been previously hospitalized in Hospital A. Overall, 15 cases were admitted to the surgical ICU in Hospital B at the time of LRSE detection, while 6 were located in a surgical ward. The earliest LRSE detection occurred in March 2023 (Pt01) in the surgery ward of Hospital B, however, this isolate fell outside the cluster threshold of 6 AD, showing a distance of 7 AD.

**Figure 2:**
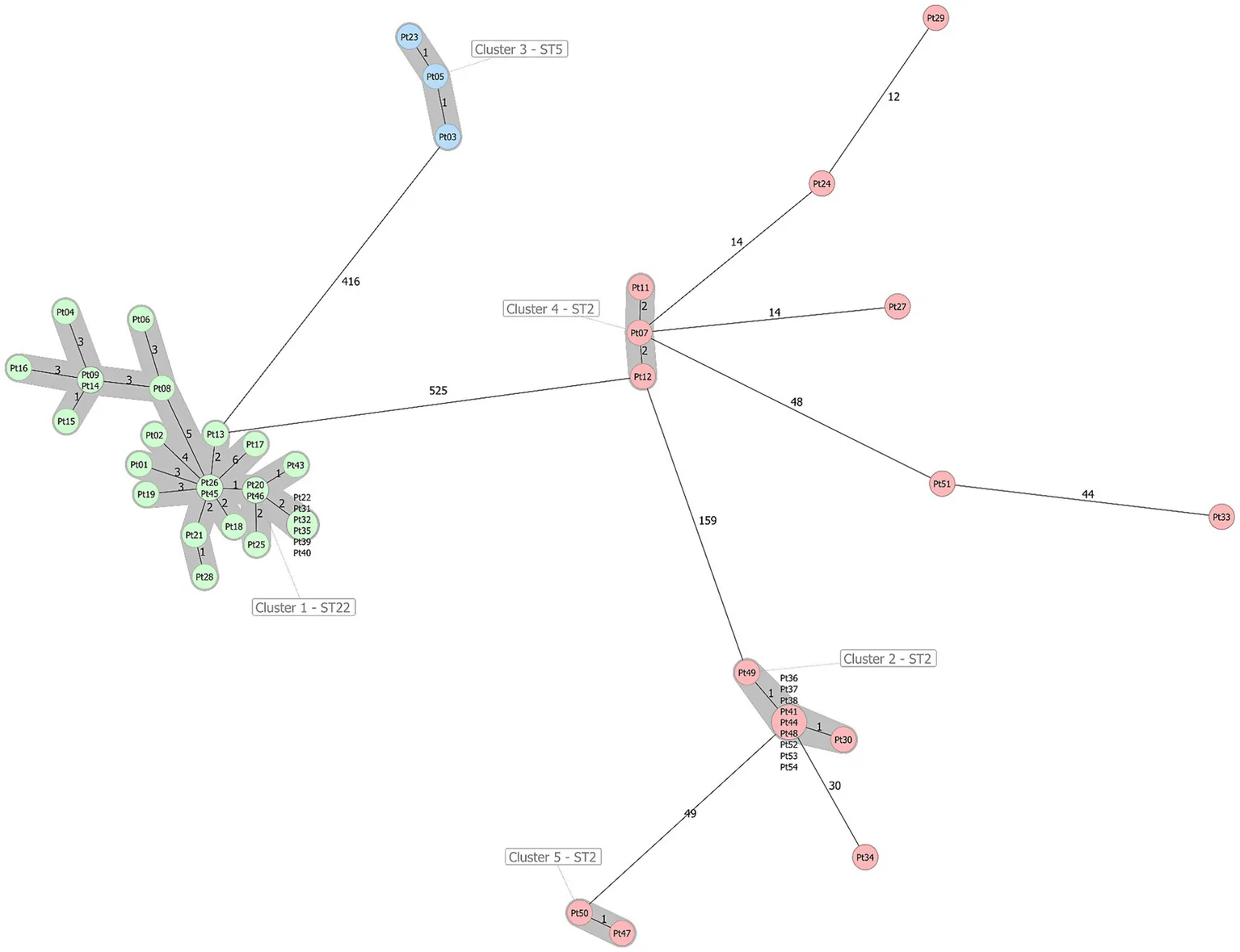
Minimum spanning tree (MST) showing the genetic relatedness between the LRSE isolates (*n* = 52). Colors indicate STs: green = ST22; blue = ST5; pink = ST2. The number of allelic differences is displayed on the connection lines. Clusters are highlighted in grey. The cluster threshold was set at six allelic differences. Short-read Illumina data were used.

Cluster 2 (ST2) consisted of 11 screening isolates obtained in Hospital A and the first detection was in October 2024. Two cases were transferred from Hospital B, while two others were transferred from two other hospitals. All but one were oncological patients staying at any of the oncology wards or visiting the outpatient oncological ward. The cluster spanned six months and isolates differed between 0 to 1 AD. Two cases of the cluster had a history of LZD administration of an unknown duration.

Cluster 3 (ST5) grouped only three patient isolates obtained in Hospital A. The first case was detected in March 2023 and last case in July 2024. All were oncological patients and there were no documented transfers to Hospital B. The cluster spanned 1.5 years and isolates differed by 2 AD. One case of the cluster had undergone LZD therapy for 9 days.

Cluster 4 (ST2) consisted of three clinical isolates from Hospital A, although the last identified case had been transferred from Hospital B. All were cancer patients that had undergone LZD therapy (9–14 days). The cluster spanned one month (June–July 2023) and isolates differed between 2 and 3 AD.

Last, Cluster 5 (ST2) included two patient isolates from Hospital B, and one of them had stayed at the surgical ICU. The cluster spanned two weeks and isolates differed by 3 AD.

Comparative analysis with publicly available LRSE genomes (*n* = 89) from previously published studies in Austria, France, and Italy revealed varying degrees of relatedness to the clusters identified in our study (Supplementary Figure S2). The closest related ST5 isolate from the Austrian 2019 study differed by 13 AD from the isolates belonging to Cluster 3. Similarly, the nearest ST2 isolate from the same study differed by 19 AD from the Cluster 5 isolates. In contrast, the Italian isolates were more distantly related, showing a separation of 41 AD from Cluster 2 (ST2) isolates and 53 AD from Cluster 4 (ST2) isolates. The French isolates exhibited an intermediate level of relatedness. The closest French isolate differed by 16 AD from Cluster 1 (ST22) isolates. Among ST2 isolates, the nearest French isolate was separated by 61 AD from Cluster 4 and by 49 AD from Cluster 5.

### 3.4 ARGs, VGs, and plasmid analysis

We found cluster-specific point mutations in the 23S rRNA region (Table 2). All Cluster 1 isolates (ST22) presented the C2534U (C2560U) and theU2504A (U2530A) mutation. Analysis of a representative hybrid assembly from Cluster 1 identified five copies of the 23S rRNA gene, four of which carried the U2504A and C2534U mutations.

**Table 2:** SNPs detected in the 23S rRNA (Domain V, *E. coli* numbering) and the 50S ribosomal proteins L3 and L4 in this study.

Location	Mutation*	Cluster and ST
23S rRNA	C2534U (C2560U), U2504A (U2530A)	Cluster 1-ST22
	G2576U (G2602U)	Cluster 2-ST2, Cluster 4-ST2, Cluster 5-ST2
L3 Protein	G152D, D159Y	Cluster 1-ST22
	M156T	Cluster 2-ST2 and LRSH
	H146Q, V154L, A157R	Cluster 3-ST5
	F147L	Cluster 4-ST2
	V154L	Cluster 5-ST2 and LRSH
L4 Protein	N158S	Cluster 1-ST22 and Cluster 3-ST5
	71-GGU-72 insertion	Cluster 3-ST5 and Cluster 4-ST2

*Mutation positions are reported according to the *E. coli* 23S rRNA numbering system, with the corresponding *S. epidermidis* positions indicated in parentheses.

Cluster 2, Cluster 4, Cluster 5 isolates (all ST2), as well as the six ST2 singletons, presented the G2576U (G2602U) mutation. Analysis of a representative ST2 hybrid genome assembly identified five 23S rRNA copies were mutated with G2576U.

In the L3 protein mutations were also detected leading to an aminoacidic change and were cluster-specific except for V154L, which was present in both Cluster 3 and Cluster 5 isolates. Regarding the L4 protein, the N158S mutation was detected in Cluster 1-ST22 and Cluster 3-ST5, whereas the 71-GGU-72 insertion, which also resulted in amino acid changes, was found in Cluster 3-ST5 and Cluster 4-ST2. Cluster 2-ST2 showed no mutations in L4. No mutations were found in L22 for any of the isolates. As for the LRSH, it presented two mutations in L3 (V154L and M156T) and a MIC of 64 mg/L.

LZD- associated transferable ARGs such as *cfr*, *cfr(D), optr*A and *pox*t were not found. We, however, detected the presence of genetic resistances other than LZD resistance (Figure 3). All LRSE isolates and the LRSH isolate carried the *me*cA cassette conferring methicillin resistance. In addition, the *qacA* gene, responsible for resistance to quaternary ammonium compounds, was detected in 48 LRSE isolates. It was found in all ST2 isolates and in all but one ST22 isolate, whereas *qacB/C* was detected in four isolates, including the three ST5 isolates and one ST2 isolate.

**Figure 3:**
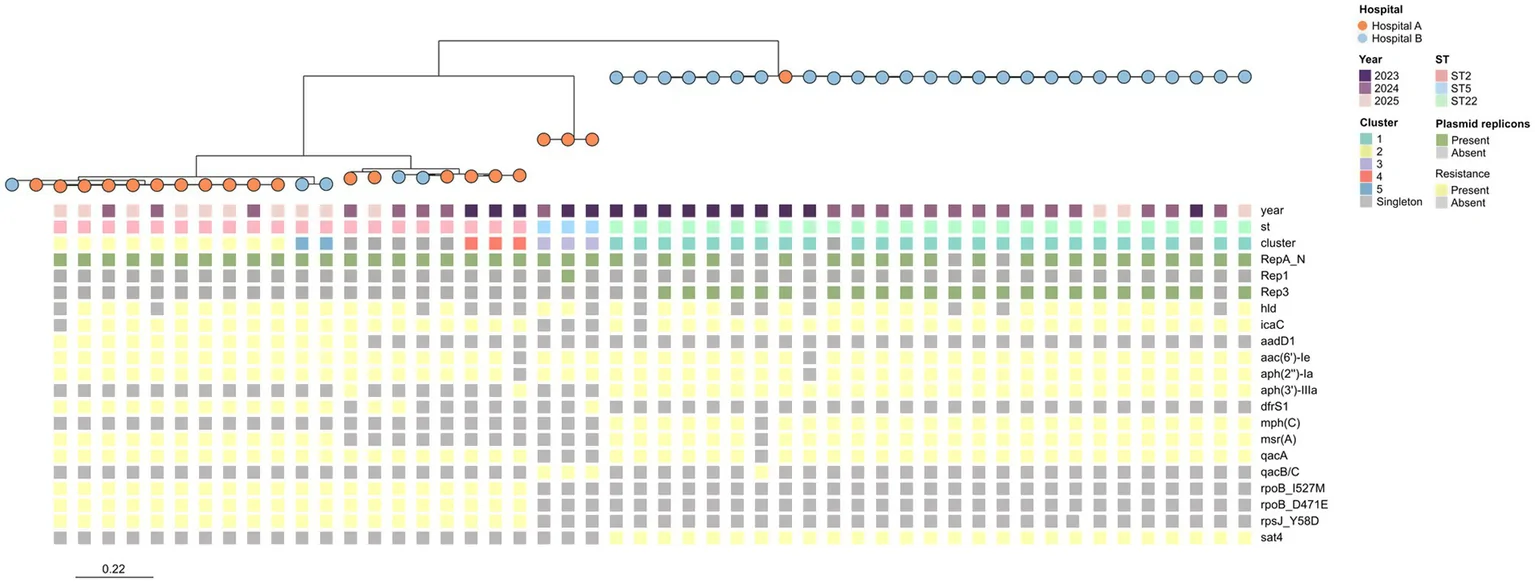
Neighbor-joining tree based on cgMLST allelic distance matrix of the 52 LRSE isolates, colored by hospital (orange: Hospital A, blue: Hospital B) and annotated by year, ST, cluster, plasmid replicons, point mutations and ARGs conferring resistance to antibiotics other than linezolid.

Genes associated with erythromycin resistance were highly prevalent: *msr(A)* was identified in 40 isolates, and *mph(C)* was found in 26 isolates. Mutations in *rpoB* (D471E and I527M) conferring rifampin resistance were detected in 22 isolates. A point mutation associated with tetracycline, tigecycline and eravacycline resistance in certain VRE (rpsJ_Y58D) was detected in 22 isolates. Trimethoprim resistance mediated by *dfrS1* was observed in 17 isolates.

Regarding virulence factors, the *ica* operon, responsible for biofilm formation, was found in all ST2 and ST22 isolates except for two, whereas it was absent in all ST5 isolates.

Plasmid replicons detected using Illumina data included RepA_N, Rep1 and Rep3. Most Hospital B isolates carried RepA_N and Rep3 (*n* = 18) while most Hospital A isolates carried RepA_N only (*n* = 19).

Nanopore sequencing for the selected isolates confirmed that no plasmids contained LZD ARGs.

### 3.5 Control measures

Following the detection of several LRSE isolates from clinical specimens and the suspicion of nosocomial transmission, active screening was implemented for all patients admitted to the most affected wards at the beginning in October 2024. Prior to implementation of the screening program, case detection relied primarily on clinical specimens obtained for diagnostic purposes. Control measures included the implementation of structured microbiological screening for LRSE upon admission to the most severely affected wards and strict isolation of affected patients. Furthermore, enhanced contact precautions, including mandatory glove and gown use when caring for LRSE-positive patients, were implemented.

## 4 Discussion

The dissemination of LZD resistance remains a major concern worldwide, particularly due to the critical role of LZD as a last-resort treatment option. In this context, our screening algorithm based on a chromogenic screening media was a reliable and convenient approach to effectively detect LRSE, as previously already shown for different clinical materials (Girlich et al., 2022).

WGS using the previously published *S. epidermidis* cgMLST scheme identified five distinct LRSE genomic clusters over the study period (2023–2025), each defined by ≤6 allelic differences. These clusters represented separate genomic lineages, clearly differentiated from one another. Although the investigation was initially triggered by simultaneous LRSE detections in the two hospitals, WGS revealed five genetically distinct clusters. These findings indicate the presence of several unrelated genomic lineages rather than a single clonal outbreak. However, the available epidemiological information was limited to the hospital, date of detection, and ward at the time of identification. Data on prior patient movements, contacts between patients, healthcare worker interactions, shared equipment, or environmental reservoirs were unavailable. Therefore, genomic clustering should be interpreted as evidence of genetic relatedness and potential epidemiological links rather than proof of direct transmission events or transmission direction. Cluster 1 (ST22) isolates displayed the widest allelic diversity within our dataset (0–6 AD) and remained present over an extended period (March 2023 to February 2025), a pattern consistent with a long-standing endemic lineage circulating in Hospital B. However, several isolates obtained in July and December 2024 (Pt22, Pt31, Pt35, Pt39 and Pt40) were genetically indistinguishable (0 AD) and were all recovered from patients in the ICU, indicating a possible recent transmission episode or a common source within this ward. In addition, the identification of a single case in Hospital A with documented diagnostic procedures in Hospital B prior to LRSE detection was consistent with Hospital B being involved in the dissemination of this cluster. However, the available epidemiological data do not allow inference of the direction of transmission or confirmation of the origin of the cluster.

In contrast, Cluster 2 (ST2) was extremely homogeneous (0–1 alleles) and concentrated within a six-month period (October 2024 to April 2025), including three isolates obtained on the same day, strongly supporting recent and ongoing transmission in an oncology ward. However, because active surveillance screening began in October 2024 and all isolates in this cluster were identified through screening, we cannot exclude the possibility that some colonizations were acquired prior to the start of the screening period. Cluster 3 (ST5) exhibited minimal allelic variation (2 AD) but isolates were separated by longer intervals (minimum of 42 days), consistent with persistence or repeated introduction in an oncology and transplantation ward. As for cluster 4 (ST2), it appeared as a shorter punctual event in summer 2023 also with limited allelic variation (2 to 3 AD) in oncology and transplantation ward. Similarly, Cluster 5 (ST2) isolates were close to each other (3 AD) and the event appeared as short (15-day window). Although three clusters were ST2, their allelic distances and temporal patterns demonstrated independent evolutionary trajectories.

Moreover, isolates involved in Clusters 1 and 2 (ST22 and ST2) were *ica*-positive, consistent with their potential biofilm-forming capacity (Becker et al., 2014). However, as *ica* positivity was also observed among non-clustered isolates of the same STs, our data did not support a specific association between *ica* carriage and sustained transmission.

LRSE surveillance ended in April 2025, coinciding with the cessation of isolate submission rather than with the resolution of transmission; clinical cases continued to occur thereafter, and infection prevention and control (IPC) measures remained in place, although their precise scope and implementation timeline were not documented. Consequently, the apparent absence of further LRSE detections after the implementation of IPC measures cannot be interpreted as evidence of healthcare-associated dissemination, nor as evidence that the measures were effective, as this apparent decline may simply reflect the end of the surveillance window. Because the investigation was observational and detailed epidemiological data were not available, a causal relationship between the implemented measures and the observed trend in LRSE detections cannot be established, and the contribution of specific transmission routes could not be determined.

Furthermore, comparison with publicly available LRSE genomes from other European countries indicated that the clusters identified in our study were genetically more closely related to isolates reported from Austria than to those from France or Italy and shows that the isolates identified in our study belong to lineages that are not restricted to a single institution (Huber et al., 2021).

While the genetic distances observed between the isolates in this study and the other three studies do not support recent transmission events, they are consistent with the long-term persistence and wider dissemination of successful hospital-adapted *S. epidermidis* clones. Nevertheless, without epidemiological data linking the respective healthcare settings, the significance of these genomic relationships should be interpreted with caution.

Most of the investigated isolates belonged to ST22 and lacked the G2576U mutation. Instead, ST22 isolates were characterized by the U2504A and C2534U mutations and exhibited significantly higher linezolid MICs than ST2 and ST5 isolates, consistent with previous reports describing highly linezolid-resistant ST22 lineages (Freitas et al., 2018). In the representative ST22 isolate, these mutations were present in four of five 23S rRNA gene copies, whereas G2576U was detected in all five copies of the representative ST2 isolate. Despite complete fixation of G2576U, ST2 isolates generally displayed lower and more variable MICs than ST22 isolates, suggesting that resistance levels are influenced not only by the proportion of mutated 23S rRNA gene copies but also by the specific mutations involved and their interaction with lineage-associated alterations in the ribosomal proteins L3 and L4.

Although a systematic assessment of *rrn* operon dosage was not performed for all isolates, our findings suggest that linezolid resistance arose through distinct evolutionary pathways across sequence types. In addition to ST-specific 23S rRNA mutations, characteristic amino acid substitutions in the 50S ribosomal proteins L3 and L4 were identified, all of which have been reported previously (Lépine et al., 2024; Dortet et al., 2018; Freitas et al., 2018).

Together, these observations indicate that resistance phenotypes likely result from specific combinations of target-site modifications rather than a single resistance mechanism. However, the individual contribution of these mutations to resistance levels, bacterial fitness, persistence, and transmission could not be determined.

Regarding the LRSH isolate, two amino acid substitutions, V154L and M156T, were identified in the L3 ribosomal protein. M156T had previously been described in LRSH, whereas V154L has been reported in other linezolid-resistant staphylococci, including *S. epidermidis* (de Almeida et al., 2013; Tewhey et al., 2014).

Regarding *qacA*, it was detected in all ST2 isolates and in all but one ST22 isolate, whereas *qacB/C* was detected in the three ST5 isolates and one ST2 isolate, suggesting a lineage-associated distribution of antiseptic tolerance determinants. The high prevalence of *qacA* is noteworthy because this gene has been associated with reduced susceptibility to chlorhexidine and other antiseptics commonly used in healthcare settings. Although genes of the *qac* family have been associated with reduced susceptibility to quaternary ammonium compounds and chlorhexidine, routine chlorhexidine bathing was not practiced at either participating hospital. Therefore, the contribution of these determinants to persistence or transmission of the investigated lineages could not be evaluated.

In addition, information on prior LZD therapy was unavailable for LRSE cases from Hospital B and it could not be confirmed whether any had previously received LZD treatment. Consequently, the role of LZD selective pressure in the emergence and spread of resistance could not be assessed, and no causal relationship between LZD exposure, particularly for Hospital B cases, and the occurrence of LRSE could be inferred from our data. This is particularly relevant, as LZD use has previously been linked to the development and dissemination of LZD resistance in hospital settings (Weßels et al., 2018). Nevertheless, published evidence indicates that up to 55% of LRSE cases occur in patients without prior exposure to LZD (Huber et al., 2021). In addition, the identification of several clusters of closely related LRSE isolates in our study suggests that transmission of already resistant strains may have contributed to the observed epidemiology. Similar findings have been reported previously ICU outbreaks globally, where a clonally related LRSE strain spread among patients and was also recovered from the hospital environment, supporting a healthcare-associated transmission (Weßels et al., 2018; Papan et al., 2021; Kelly et al., 2008; Treviño et al., 2009). However, the relative contributions of clonal dissemination and resistance emergence under antimicrobial selection pressure in our study could not be evaluated. Therefore, while the observed clustering is consistent with healthcare-associated transmission, the respective roles of transmission and antimicrobial selection remain uncertain.

A further limitation concerns the interpretation of cgMLST clustering. The ≤6-allele threshold used in this study was adopted from previous work on LRSE (Campmann et al., 2023) but has not been specifically validated for the STs or patient population included here. Consequently, cluster assignment should be interpreted as an indicator of close genomic relatedness and possible epidemiological linkage rather than definitive evidence of direct transmission.

In summary, our findings indicate that LRSE isolates from the two hospitals belonged to five genetically distinct clusters. Cluster 1 was predominantly detected in Hospital B, particularly in the surgical ICU, whereas Clusters 2–4 were identified exclusively in Hospital A. However, the available epidemiological information was insufficient to determine the origin of individual clusters or reconstruct transmission pathways. Rather than representing a single clonal event, the LRSE isolates belonged to multiple genetically distinct lineages circulating during the study period. These findings highlight the importance of combining genomic surveillance with comprehensive epidemiological investigations to better understand and control the spread of LRSE in healthcare settings.

## Data Availability

The sequencing data (both Illumina and Nanopore) produced in this study have been submitted to the Sequence Read Archive (SRA) and are publicly available under the BioProject accession number PRJNA1492009.

## Supplementary material

SUPPLEMENTARY TABLE S1 Demographic and clinical metadata of the 53 LRSE-positive patients.

SUPPLEMENTARY TABLE S2 Distinctive genetic and phenotypic features of the 53 LRSE isolates investigated in this study.

SUPPLEMENTARY FIGURE S1 LZD MIC distribution among the 52 successfully sequenced LRSE Isolates, by sequence type (ST).

SUPPLEMENTARY FIGURE S2 Minimum spanning tree (cgMLST) showing genomic relationships between international LRSE isolates (*n* = 89) and successfully sequenced isolates from this study (*n* = 52).
